# Redetermination of β-Ba(PO_3_)_2_

**DOI:** 10.1107/S1600536814000361

**Published:** 2014-01-11

**Authors:** Matthias Weil

**Affiliations:** a Institute for Chemical Technologies and Analytics, Division of Structural Chemistry, Vienna University of Technology, Getreidemarkt 9/164-SC, A-1060 Vienna, Austria

## Abstract

In comparison with the previous structure determination of the β-modification of barium *catena*-polyphosphate that was based on Weissenberg film data [Grenier *et al.* (1967 ▶). *Bull. Soc. Fr. Minéral. Cristallogr.* **90**, 24–31], the current CCD-data-based redetermination reveals all atoms with anisotropic displacement parameters, standard uncertainties for the atomic coordinates, and the determination of the absolute structure. Moreover, a much higher accuracy in terms of the bond-length distribution for the polyphosphate chain, with two shorter and two longer P—O distances, was achieved. The structure consists of polyphosphate chains extending parallel to [100] with a periodicity of two PO_4_ tetra­hedra. The Ba^2+^ cations are located between the chains and are surrounded by ten O atoms in the form of a distorted coordination polyhedron, with Ba—O distances ranging from 2.765 (3) to 3.143 (3) Å, also reflecting the higher precision of the current redetermination.

## Related literature   

For polymorphism of Ba(PO_3_)_2_, see: Grenier & Martin (1975 ▶). For the previous structure refinement of β-Ba(PO_3_)_2_, see: Grenier *et al.* (1967 ▶). For the structure refinement of γ-Ba(PO_3_)_2_, see: Coing-Boyat *et al.* (1978 ▶). For the crystal chemistry of condensed phosphates, see: Durif (1995 ▶). For standardization of structure data, see: Gelato & Parthé (1987 ▶).

## Experimental   

### 

#### Crystal data  

Ba(PO_3_)_2_
*M* *_r_* = 295.28
Orthorhombic, 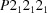

*a* = 4.4979 (2) Å
*b* = 8.3377 (4) Å
*c* = 13.3911 (6) Å
*V* = 502.19 (4) Å^3^
*Z* = 4
Mo *K*α radiation
μ = 8.50 mm^−1^
*T* = 293 K
0.15 × 0.08 × 0.05 mm

#### Data collection  

Bruker SMART CCD diffractometer
Absorption correction: multi-scan (*SADABS*; Bruker, 2007 ▶) *T*_min_ = 0.362, *T*_max_ = 0.676
5836 measured reflections
1587 independent reflections
1560 reflections with *I* > 2σ(*I*)
*R*_int_ = 0.016

#### Refinement  

*R*[*F* ^2^ > 2σ(*F* ^2^)] = 0.020
*wR*(*F* ^2^) = 0.047
*S* = 1.13
1587 reflections
82 parameters
Δρ_max_ = 1.09 e Å^−3^
Δρ_min_ = −0.75 e Å^−3^
Absolute structure: Flack (1983 ▶), 624 Friedel pairs
Absolute structure parameter: 0.04 (2)

### 

Data collection: *SMART* (Bruker, 2007 ▶); cell refinement: *SAINT* (Bruker, 2007 ▶); data reduction: *SAINT*; program(s) used to solve structure: *SHELXS97* (Sheldrick, 2008 ▶); program(s) used to refine structure: *SHELXL97* (Sheldrick, 2008 ▶); molecular graphics: *ATOMS* (Dowty, 2006 ▶); software used to prepare material for publication: *publCIF* (Westrip, 2010 ▶).

## Supplementary Material

Crystal structure: contains datablock(s) I, global. DOI: 10.1107/S1600536814000361/pj2008sup1.cif

Structure factors: contains datablock(s) I. DOI: 10.1107/S1600536814000361/pj2008Isup2.hkl

CCDC reference:

Additional supporting information:  crystallographic information; 3D view; checkCIF report

## Figures and Tables

**Table 1:** Comparison of the P—O bond lengths (Å) of the two related PO_4_ tetra­hedra in the current and the previous (Grenier *et al.*, 1967 ▶) refinement of β-Ba(PO_3_)_2_

current refinement	previous refinement
P1—O3 1.475 (2)^viii^	P2—O6 1.427
P1—O1 1.480 (2)^viii^	P2—O5 1.540
P1—O6 1.607 (2)^ix^	P2—O2 1.590
P1—O4 1.625 (2)^viii^	P2—O1 1.652
P2—O2 1.481 (2)	P1—O3 1.476
P2—O5 1.486 (2)^vii^	P1—O4 1.540
P2—O6 1.604 (3)^vi^	P1—O2 1.559
P2—O4 1.607 (3)^vi^	P1—O1 1.621

Symmetry codes: (vi) −*x* + 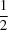, −*y*, *z* + 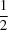; (vii) *x* − 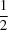, −*y* + 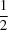, −*z* + 1; (viii) −*x*, *y* + 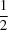, −*z* + 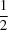; (ix) −*x* + 1, *y* + 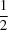, −*z* + 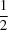; (*x*) *x* − 1, *y*, *z*.

**Fig. 1.**
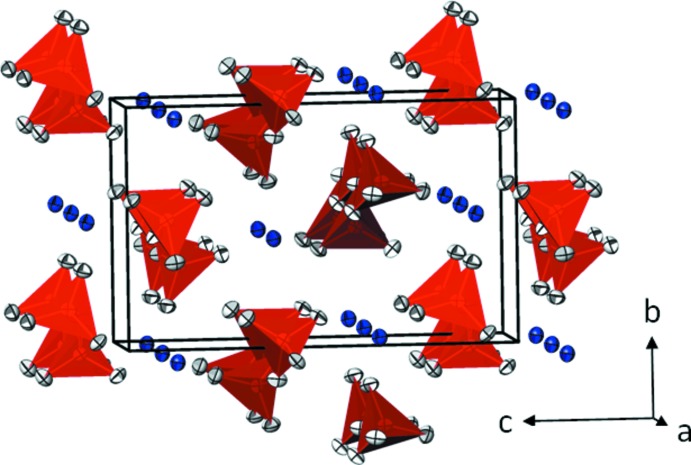
The crystal structure of β-Ba(PO3)2 in a projection approximately along [100]. Ba2+ cations are shown in blue, PO4 tetrahedra in red with O atoms in white. Displacement ellipsoids are drawn at the 90% probability level. For clarity, no Ba—O bonds are shown.

**Fig. 2.**
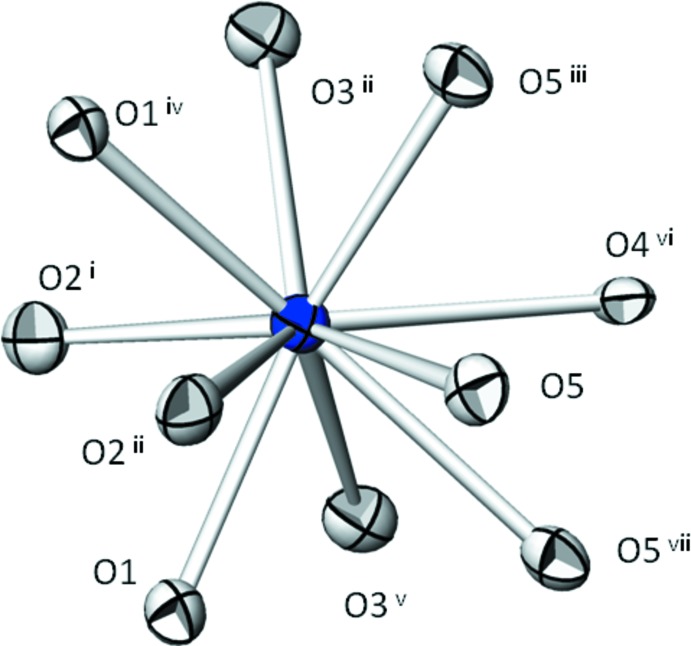
The irregular BaO10 coordination polyhedron in the structure of β-Ba(PO3)2. Displacement ellipsoids are drawn at the 90% probability level. [Symmetry codes: (i) -x + 1/2, -y, z - 1/2; (ii) -x + 1, y - 1/2, -z + 1/2; (iii) x + 1/2, -y + 1/2, -z + 1; (iv) x + 1, y, z; (v) -x, y - 1/2, -z + 1/2; (vi) -x + 1/2, -y, z + 1/2; (vii) x - 1/2, -y + 1/2, -z + 1.]

## supplementary crystallographic information

### 1. Comment

Polymorphism of Ba(PO_3_)_2_ with three modifications has been reported by Grenier & Martin (1975): The stable β-form transforms to the high-temperature *α*-form at 1058 K, and the *γ*-form transforms at 978 K to the β-form. Structure determinations were carried out for the *γ*-form (Coing-Boyat *et al.*, 1978) and for the β-form (Grenier *et al.*, 1967). The crystal structure of α-Ba(PO_3_)_2_ is yet unknown. Comparative discussions of the structural set-up of the β- and *γ*-form of Ba(PO_3_)_2_ and of other divalent long-chain polyphosphates were given by Durif (1995).

During experiments intended to isolate crystals of *α*-Ba(PO_3_)_2_ by quenching the reaction product from the re-crystallized melt at temperatures above the indicated transition point, high-quality crystals of β-Ba(PO_3_)_2_ were obtained instead. Since the first structure refinement of this modification was based on Weissenberg film data and converged with a relatively high residual *R* = 0.1, with atoms refined only with isotropic displacement factors and without indication of standard uncertainties for the fractional atomic coordinates, a re-refinement of the structure with modern CCD-based data seemed appropriate. The results of this re-refinement are reported here, confirming in principle the results of Grenier *et al.* (1967), however, achieving bond lengths and angles with much higher accuracy and precision, as exemplified by a comparison of the P—O bond length (Table 1).

The *catena*-polyphosphate chain has a periodicity of two PO_4_ tetrahedra and extends parallel to [100] (Fig. 1). In comparison with the previous structure refinement (Grenier *et al.*, 1967), the determined bond lengths of the present refinement are in much better agreement with the usually observed bond length distribution in such long-chain polyphosphates (Durif, 1995), with two shorter and two longer P—O distances, each with similar values (Table 1).

The Ba^2+^ cation is located between the chains and is surrounded by ten oxygen atoms in an irregular coordination sphere with Ba—O distances in the range from 2.765 (3) to 3.143 (3) Å (Fig. 2).

### 2. Experimental

Stoichiometric amounts of BaCO_3_ and (NH_4_)_2_HPO_4_ (molar ratio 1:2) with a 3% excess of the phosphate precursor were finely ground, heated in a platinum crucible to 1173 K and slowly cooled to 1073 K at a rate of 2 K h^-1^. Then the crucible was quenched in a cold water bath. Colourless fragments of the title compound were cut from the clear, transparent reaction product.

### 3. Refinement

In contrast to the previous structure refinement (Grenier *et al.*, 1967) with *a* = 4.510 (2), *b* = 13.44 (2) *c* = 8.36 (5) Å, the reduced cell setting was chosen for the current refinement. Structure data were finally standardized with *STRUCTURE-TIDY* (Gelato & Parthé, 1987). The highest and lowest remaining electron densities are located 0.75 Å from Ba and 1.19 Å from O6, respectively.

### Crystal data

**Table:**

Ba(PO_3_)_2_	*F*(000) = 536
*M**_r_* = 295.28	*D*_x_ = 3.905 Mg m^−^^3^
Orthorhombic, *P*2_1_2_1_2_1_	Mo *K*α radiation, λ = 0.71073 Å
Hall symbol: P 2ac 2ab	Cell parameters from 4713 reflections
*a* = 4.4979 (2) Å	θ = 2.4–31.0°
*b* = 8.3377 (4) Å	µ = 8.50 mm^−^^1^
*c* = 13.3911 (6) Å	*T* = 293 K
*V* = 502.19 (4) Å^3^	Fragment, colourless
*Z* = 4	0.15 × 0.08 × 0.05 mm

### Data collection

**Table:**

Bruker SMART CCD diffractometer	1587 independent reflections
Radiation source: fine-focus sealed tube	1560 reflections with *I* > 2σ(*I*)
Graphite monochromator	*R*_int_ = 0.016
ω scan	θ_max_ = 31.0°, θ_min_ = 2.9°
Absorption correction: multi-scan (*SADABS*; Bruker, 2007)	*h* = −6→6
*T*_min_ = 0.362, *T*_max_ = 0.676	*k* = −12→11
5836 measured reflections	*l* = −19→19

### Refinement

**Table:**

Refinement on *F*^2^	Primary atom site location: structure-invariant direct methods
Least-squares matrix: full	Secondary atom site location: difference Fourier map
*R*[*F*^2^ > 2σ(*F*^2^)] = 0.020	*w* = 1/[σ^2^(*F*_o_^2^) + (0.0223*P*)^2^ + 0.9553*P*] where *P* = (*F*_o_^2^ + 2*F*_c_^2^)/3
*wR*(*F*^2^) = 0.047	(Δ/σ)_max_ = 0.001
*S* = 1.13	Δρ_max_ = 1.09 e Å^−^^3^
1587 reflections	Δρ_min_ = −0.75 e Å^−^^3^
82 parameters	Absolute structure: Flack (1983), 624 Friedel pairs
0 restraints	Absolute structure parameter: 0.04 (2)

### Special details

**Table:**

Geometry. All e.s.d.'s (except the e.s.d. in the dihedral angle between two l.s. planes) are estimated using the full covariance matrix. The cell e.s.d.'s are taken into account individually in the estimation of e.s.d.'s in distances, angles and torsion angles; correlations between e.s.d.'s in cell parameters are only used when they are defined by crystal symmetry. An approximate (isotropic) treatment of cell e.s.d.'s is used for estimating e.s.d.'s involving l.s. planes.
Refinement. Refinement of *F*^2^ against ALL reflections. The weighted *R*-factor *wR* and goodness of fit *S* are based on *F*^2^, conventional *R*-factors *R* are based on *F*, with *F* set to zero for negative *F*^2^. The threshold expression of *F*^2^ > σ(*F*^2^) is used only for calculating *R*-factors(gt) *etc*. and is not relevant to the choice of reflections for refinement. *R*-factors based on *F*^2^ are statistically about twice as large as those based on *F*, and *R*- factors based on ALL data will be even larger.

### Fractional atomic coordinates and isotropic or equivalent isotropic displacement parameters (Å^2^)

**Table:**

	*x*	*y*	*z*	*U*_iso_*/*U*_eq_	
Ba	0.49023 (4)	0.05203 (2)	0.375501 (12)	0.01202 (6)	
P1	0.03622 (19)	0.67382 (10)	0.34674 (6)	0.00958 (15)	
P2	0.04126 (18)	0.03118 (10)	0.60393 (6)	0.01002 (15)	
O1	0.0024 (8)	0.1293 (3)	0.25946 (16)	0.0142 (4)	
O2	0.0272 (6)	0.1222 (3)	0.69880 (17)	0.0153 (4)	
O3	0.0307 (6)	0.3369 (3)	0.11686 (17)	0.0149 (4)	
O4	0.1343 (5)	0.0429 (3)	0.08393 (18)	0.0116 (4)	
O5	0.4603 (6)	0.3816 (3)	0.48895 (17)	0.0142 (5)	
O6	0.6315 (5)	0.1360 (3)	0.11585 (19)	0.0131 (4)	

### Atomic displacement parameters (Å^2^)

**Table:**

	*U*^11^	*U*^22^	*U*^33^	*U*^12^	*U*^13^	*U*^23^
Ba	0.01116 (8)	0.01345 (9)	0.01147 (8)	−0.00007 (7)	−0.00044 (8)	0.00042 (6)
P1	0.0082 (3)	0.0093 (3)	0.0113 (3)	0.0002 (3)	−0.0007 (3)	−0.0001 (3)
P2	0.0087 (3)	0.0103 (3)	0.0111 (3)	0.0000 (3)	−0.0001 (3)	0.0008 (3)
O1	0.0152 (9)	0.0156 (10)	0.0118 (9)	0.0018 (12)	0.0002 (11)	0.0016 (8)
O2	0.0163 (11)	0.0151 (10)	0.0145 (10)	−0.0008 (11)	−0.0010 (10)	−0.0037 (8)
O3	0.0167 (11)	0.0110 (10)	0.0171 (10)	−0.0020 (10)	−0.0019 (12)	0.0017 (8)
O4	0.0076 (9)	0.0135 (11)	0.0137 (11)	0.0021 (9)	−0.0010 (8)	−0.0047 (9)
O5	0.0156 (13)	0.0129 (10)	0.0140 (10)	−0.0012 (10)	0.0022 (10)	0.0041 (8)
O6	0.0072 (9)	0.0118 (10)	0.0203 (12)	−0.0004 (8)	−0.0031 (9)	−0.0014 (10)

### Geometric parameters (Å, º)

**Table:**

Ba—O1	2.765 (3)	Ba—O5	3.143 (3)
Ba—O2^i^	2.778 (2)	P1—O3^viii^	1.475 (2)
Ba—O3^ii^	2.806 (2)	P1—O1^viii^	1.480 (2)
Ba—O5^iii^	2.841 (3)	P1—O6^ix^	1.607 (2)
Ba—O1^iv^	2.852 (3)	P1—O4^viii^	1.625 (2)
Ba—O2^iii^	2.897 (2)	P2—O2	1.481 (2)
Ba—O3^v^	2.952 (2)	P2—O5^vii^	1.486 (2)
Ba—O4^vi^	2.955 (2)	P2—O6^vi^	1.604 (3)
Ba—O5^vii^	3.047 (3)	P2—O4^vi^	1.607 (3)
			
O1—Ba—O2^i^	67.71 (8)	O3^ii^—Ba—O5	125.05 (7)
O1—Ba—O3^ii^	141.17 (7)	O5^iii^—Ba—O5	63.43 (6)
O2^i^—Ba—O3^ii^	73.64 (7)	O1^iv^—Ba—O5	95.77 (7)
O1—Ba—O5^iii^	154.74 (7)	O2^iii^—Ba—O5	49.37 (6)
O2^i^—Ba—O5^iii^	131.84 (8)	O3^v^—Ba—O5	118.72 (7)
O3^ii^—Ba—O5^iii^	61.94 (7)	O4^vi^—Ba—O5	76.68 (7)
O1—Ba—O1^iv^	106.39 (7)	O5^vii^—Ba—O5	61.30 (6)
O2^i^—Ba—O1^iv^	71.14 (7)	O3^viii^—P1—O1^viii^	121.65 (14)
O3^ii^—Ba—O1^iv^	62.86 (7)	O3^viii^—P1—O6^ix^	105.50 (14)
O5^iii^—Ba—O1^iv^	72.72 (7)	O1^viii^—P1—O6^ix^	111.06 (17)
O1—Ba—O2^iii^	68.54 (8)	O3^viii^—P1—O4^viii^	109.50 (15)
O2^i^—Ba—O2^iii^	101.50 (3)	O1^viii^—P1—O4^viii^	108.97 (15)
O3^ii^—Ba—O2^iii^	124.60 (7)	O6^ix^—P1—O4^viii^	97.43 (13)
O5^iii^—Ba—O2^iii^	89.67 (7)	O2—P2—O5^vii^	117.17 (15)
O1^iv^—Ba—O2^iii^	63.57 (7)	O2—P2—O6^vi^	109.83 (14)
O1—Ba—O3^v^	62.04 (7)	O5^vii^—P2—O6^vi^	112.95 (15)
O2^i^—Ba—O3^v^	71.91 (7)	O2—P2—O4^vi^	112.26 (15)
O3^ii^—Ba—O3^v^	102.70 (7)	O5^vii^—P2—O4^vi^	105.74 (15)
O5^iii^—Ba—O3^v^	133.37 (7)	O6^vi^—P2—O4^vi^	97.02 (13)
O1^iv^—Ba—O3^v^	142.84 (6)	P1^v^—O1—Ba	133.76 (18)
O2^iii^—Ba—O3^v^	128.82 (7)	P1^v^—O1—Ba^x^	119.03 (18)
O1—Ba—O4^vi^	116.29 (7)	Ba—O1—Ba^x^	106.39 (7)
O2^i^—Ba—O4^vi^	131.24 (7)	P2—O2—Ba^vi^	117.64 (13)
O3^ii^—Ba—O4^vi^	86.55 (7)	P2—O2—Ba^vii^	100.85 (12)
O5^iii^—Ba—O4^vi^	65.78 (7)	Ba^vi^—O2—Ba^vii^	141.37 (9)
O1^iv^—Ba—O4^vi^	136.74 (7)	P1^v^—O3—Ba^ix^	137.20 (14)
O2^iii^—Ba—O4^vi^	125.91 (7)	P1^v^—O3—Ba^viii^	112.75 (13)
O3^v^—Ba—O4^vi^	69.76 (7)	Ba^ix^—O3—Ba^viii^	102.70 (7)
O1—Ba—O5^vii^	70.83 (6)	P2^i^—O4—P1^v^	126.27 (15)
O2^i^—Ba—O5^vii^	125.48 (8)	P2^i^—O4—Ba^i^	103.09 (11)
O3^ii^—Ba—O5^vii^	134.05 (7)	P1^v^—O4—Ba^i^	129.06 (12)
O5^iii^—Ba—O5^vii^	99.57 (7)	P2^iii^—O5—Ba^vii^	128.45 (15)
O1^iv^—Ba—O5^vii^	156.26 (7)	P2^iii^—O5—Ba^iii^	102.60 (13)
O2^iii^—Ba—O5^vii^	94.54 (7)	Ba^vii^—O5—Ba^iii^	99.57 (7)
O3^v^—Ba—O5^vii^	57.92 (7)	P2^iii^—O5—Ba	90.73 (11)
O4^vi^—Ba—O5^vii^	48.51 (6)	Ba^vii^—O5—Ba	120.73 (9)
O1—Ba—O5	91.95 (7)	Ba^iii^—O5—Ba	114.41 (9)
O2^i^—Ba—O5	150.21 (7)	P2^i^—O6—P1^ii^	130.68 (17)

Symmetry codes: (i) −*x*+1/2, −*y*, *z*−1/2; (ii) −*x*+1, *y*−1/2, −*z*+1/2; (iii) *x*+1/2, −*y*+1/2, −*z*+1; (iv) *x*+1, *y*, *z*; (v) −*x*, *y*−1/2, −*z*+1/2; (vi) −*x*+1/2, −*y*, *z*+1/2; (vii) *x*−1/2, −*y*+1/2, −*z*+1; (viii) −*x*, *y*+1/2, −*z*+1/2; (ix) −*x*+1, *y*+1/2, −*z*+1/2; (x) *x*−1, *y*, *z*.

### Comparison of the P—O bond lengths (Å) of the two related PO_4_ tetrahedra in the current and the previous (Grenier *et al.*, 1967) refinement of β-Ba(PO_3_)_2_

**Table:**

current refinement	previous refinement
P1—O3 1.475 (2)^viii^	P2—O6 1.427
P1—O1 1.480 (2)^viii^	P2—O5 1.540
P1—O6 1.607 (2)^ix^	P2—O2 1.590
P1—O4 1.625 (2)^viii^	P2—O1 1.652
P2—O2 1.481 (2)	P1—O3 1.476
P2—O5 1.486 (2)^vii^	P1—O4 1.540
P2—O6 1.604 (3)^vi^	P1—O2 1.559
P2—O4 1.607 (3)^vi^	P1—O1 1.621

Symmetry codes: (vi) -x+1/2, -y, z+1/2; (vii) x-1/2, -y+1/2, -z+1; (viii) -x, y+1/2, -z+1/2; (ix) -x+1, y+1/2, -z+1/2; (x) x-1, y, z.
